# Supplementary material for: Using machine learning to predict judgments on Western visual art along content-representational and formal-perceptual attributes
Source: PLoS One. 2024 Sep 6;19(9):e0304285. doi: 10.1371/journal.pone.0304285 (PMC11379394; doi:10.1371/journal.pone.0304285)
Supplement: S1 Table — (PDF) [file pone.0304285.s001.pdf]

S1 Table. Scales of art-judgements (targets in machine learning analysis) used in the study, German version.

	**Anweisung**	*Bitte geben Sie Ihre Bewertung zu der Zeichnung ab:*
**Aspekte**	**Skalenpunkte für Kunsturteile**	**Frageleiste**
I. Ästhetische Aspekte	1. Ästhetisch bewegend	Wie ästhetisch bewegend empfinden Sie das Kunstwerk?
	2. Schönheit	Wie schön finden Sie das Kunstwerk?
II. Qualitative Aspekte	3. Gutes Kunstwerk	Finden Sie die Zeichnung ein gutes Kunstwerk?
	4. Kreativität	Wie kreativ finden Sie das Kunstwerk?
	5. Technische Fertigkeit	Wie hoch bewerten Sie die technische Kunstfertigkeit des Künstlers/der Künstlerin?
III. Epistemische Aspekte	6. Faszinierend (intellektuell)	Finden Sie das Kunstwerk faszinierend/intellektuell stimulierend?
	7. Interessant	Wie interessant finden Sie das Kunstwerk?
	8. Zum Nachdenken anregend	Wie sehr stimmt Sie das Kunstwerk Sie nachdenklich?
IV. Ablehnende Aspekte	9. Langweilig	Wie langweilig finden Sie das Kunstwerk?
	10. Verstörend, irritierend	Wie verstörend, irritierend empfinden Sie das Kunstwerk?
V. Generelle Aspekte	11. Bekanntheit	Wie bekannt kommt Ihnen das Kunstwerk vor?
	12. Verstehen der Bedeutung	Wie sehr verstehen Sie das Kunstwerk?
VI. Präferenzaspekte	13. Gefallen	Wie sehr gefällt Ihnen das Kunstwerk persönlich?
