# Supplementary material for: Using machine learning to predict judgments on Western visual art along content-representational and formal-perceptual attributes
Source: PLoS One. 2024 Sep 6;19(9):e0304285. doi: 10.1371/journal.pone.0304285 (PMC11379394; doi:10.1371/journal.pone.0304285)
Supplement: S2 Table — (PDF) [file pone.0304285.s002.pdf]

S2 Table. Scales of art attributes (predictors in machine learning analysis) used in the study, German version.

| | **Anweisung** | *Bitte bewerten Sie das Kunstwerk anhand der unterschiedlichen Dimensionen und Attribute:* | |
|---|---|---|---|
| **Attribute** | **Skalenpunkte für Merkmalsdimensionen** | **Negativer Pol (Minimum)** | **Positiver Pol (Maximum)** |
| **i. Formal-perzeptive Attribute** | a. Visuelle Harmonie (Balance) | visuelle Harmonie, proportional | eigenartig, seltsame Formen |
| | b. Tiefenwahrnehmung | zwei-dimensional | drei-dimensional |
| | c. Komplexität | einfach | komplex |
| | d. Farbsättigung | sanft, pastell | intensive, kräftig |
| | e. Farbvielfalt | wenige Farben | Farbvielfalt |
| | f. Farbtemperatur | warme Farben | kalte Farben |
| | g. Farbwelt | dunkle Farbwelt | helle Farbwelt |
| | h. Pinselführung | feine Pinselführung | grobe Pinselführung |
| | i. Ausnützung der Zeichenfläche | wenig Ausnützung der Malfläche | sämtliche Ausnützung der Malfläche |
| **ii. Inhaltlich-repräsentative Attribute** | j. Abstraktion | repräsentativ | abstrakt |
| | k. Imagination | realistischer/s Inhalt/ Thema | imaginär, unwirklich, fantastisch |
| | l. Symbolismus (Ambiguität) | eindeutig (klare Interpretation der Darstellung) | symbolisch (mehr Interpretationsfreiraum) |
| | m. Akkurate Objektdarstellung | fotorealistisch | malerisch |
| | n. Lebendigkeit, Animation | dynamisch | still |
| | o. Emotionalität | emotionslos | emotional aufgeladen |
| | p. Valenz | negative Stimmung | positive Stimmung |
| | q. Fokussierung | viel Kontext/Umgebung im Bild | fokussierter Inhalt |
