# Supplementary material for: Using machine learning to predict judgments on Western visual art along content-representational and formal-perceptual attributes
Source: PLoS One. 2024 Sep 6;19(9):e0304285. doi: 10.1371/journal.pone.0304285 (PMC11379394; doi:10.1371/journal.pone.0304285)
Supplement: S3 Table — ID represents the VAPS-identification code. (PDF) [file pone.0304285.s003.pdf]

S3 Table. Full list of stimulus-set rated including name of artist, title, style, and depicted motif. ID represents the VAPS-identification code.

ID	Name of Artist	Title	Style	Category
20402	Gentileschi, Orazio	Portrait of a Young Woman as a Sibyl	Baroque	Portrait
20409	Rubens, Peter Paul	Portrait of a Woman, Probably Susanna Lunden	Baroque	Portrait
20411	Vermeer, Jan	Das Maedchen mit dem Perlenohrgehnge	Baroque	Portrait
20807	Nattier, Jean-Marc	Elizabeth, Countess of Warwick	Rococo	Portrait
20809	Gainsborough, Thomas	Portrait of Mrs. Gainsborough	Rococo	Portrait
20814	Reynolds, Joshua	Self Portrait	Rococo	Portrait
21401	Degas, Edgar	Duchess of Montejasi	Impressionism	Portrait
21407	Renoir, Pierre-Auguste	Portrait of Madame Alphonse Daudet	Impressionism	Portrait
21409	Renoir, Pierre-Auguste	Hyacinthe-Eugène Meunier (1841-1906), Called Eugène Murer	Impressionism	Portrait
21602	Cezanne, Paul	Portrait of Madame Cezanne	Postimpressionism	Portrait
21609	Picasso, Pablo	Femme la Chemise	Postimpressionism	Portrait
21610	Gogh van, Vincent	Italian Woman (Agostina Segatori)	Postimpressionism	Portrait
22401	Dix, Otto	The Nun	Cubism	Portrait
22403	Metzinger, Jean	Le Go√ªter, Tea Time	Cubism	Portrait
22407	Feininger, Lyonel	Selbstbildnis	Cubism	Portrait
23005	Magritte, Rene	Man in a Bowler Hat (Man mit Melone)	Surrealism	Portrait
23006	Magritte, Rene	Clairvoyance	Surrealism	Portrait
23007	Magritte, Rene	Rape (La Violazione)	Surrealism	Portrait
30701	Everdingen van, Allaert	Swedish Landscape	Baroque	Landscape
30702	Goyen van, Jan	Landscape with Dunes	Baroque	Landscape
30709	Vermeer, Jan	View of Delft, Netherlands, after the Fire	Baroque	Landscape
30801	Canaletto/ Bellotto, Bernardo	View of Dresden from the Left Bank of the Elbe, below the Fortifications	Rococo	Landscape
30802	Canaletto/ Bellotto, Bernardo	Vienna seen from the Belvedere	Rococo	Landscape
30806	Guardi, Francesco	The Feast of Maundy Thursday in Venice	Rococo	Landscape
31405	Monet, Claude	La Gare Saint-Lazare	Impressionism	Landscape
31406	Monet, Claude	Haystacks, End of Summer, Giverny	Impressionism	Landscape
31411	Sisley, Alfred	Pradera	Impressionism	Landscape
31601	Cezanne, Paul	The Lac d'Annecy	Postimpressionism	Landscape
31603	Cezanne, Paul	Mont Sainte-Victoire	Postimpressionism	Landscape
31605	Gogh van, Vincent	Cafe Terrace at Night (Place du Forum in Arles)	Postimpressionism	Landscape
32401	Delaunay, Robert	Eiffel Tower with Trees	Cubism	Landscape
32404	Rivera, Diego	Trees and Walls in Toledo	Cubism	Landscape
32405	Marc, Franz	Kleine Komposition II (Haus mit B√§umen)	Cubism	Landscape
33003	Magritte, Rene	Blood with Tell (La voix du sang)	Surrealism	Landscape
33005	Matta, Roberto	Invasion of the Night	Surrealism	Landscape
33006	Matta, Roberto	The Onyx of Electra	Surrealism	Landscape
40702	Aertsen, Pieter	Butcher Shop	Baroque	Still life
40713	Hoogstraten van, Samuel	Slippers, Dutch Interior	Baroque	Still life
40717	Snyders, Frans	Three Monkeys with Fruit	Baroque	Still life
40801	Chardin, Jean Sim√©on	Dead Hare with Powder Flask and Game-bag	Rococo	Still life

40802	Chardin, Jean Simeon	Musical Instruments and Parrot	Rococo	Still life
40806	Vallayer-Coster, Anne	Bouquet of Flowers in a Terracotta Vase, with Peaches and Grapes	Rococo	Still life
41401	Manet, √âdouard	Bunch of Asparagus	Impressionism	Still life
41402	Monet, Claude	Apples and Grapes	Impressionism	Still life
41403	Monet, Claude	Bouquet of Sunflowers	Impressionism	Still life
41610	Gauguin, Paul	The Ham	Postimpressionism	Still life
41612	Gogh van, Vincent	Irises	Postimpressionism	Still life
41616	Gogh van, Vincent	Head of a Skeleton with a burning Cigarette	Postimpressionism	Still life
42401	Braque, Georges	Composition with Ace of Clubs	Cubism	Still life
42402	Braque, Georges	Still Life with Grapes and Clarinet	Cubism	Still life
42406	Gris, Juan	Guitar and Glasses	Cubism	Still life
43006	Magritte, Rene	Personal Values (Les valeurs personnelles)	Surrealism	Still life
43009	O'Keeffe, Georgia	Head with Broken Pot	Surrealism	Still life
43010	Ernst, Max	√ñdipus Rex	Surrealism	Still life
