# Supplementary material for: Using machine learning to predict judgments on Western visual art along content-representational and formal-perceptual attributes
Source: PLoS One. 2024 Sep 6;19(9):e0304285. doi: 10.1371/journal.pone.0304285 (PMC11379394; doi:10.1371/journal.pone.0304285)
Supplement: S4 Table — (PDF) [file pone.0304285.s004.pdf]

S4 Table. Stimulus selection of VAPS using Wilk-Shapiro test for ensuring best possible normal distribution of VAPS ratings while including images.

	**Wilk-Shapiro**	***p*-value**
	**All depicted motif/style combined**	
Liking	$W = 0.98$	$p = 0.52$
Valence	$W = 0.98$	$p = 0.54$
Arousal	$W = 0.95$	$p = 0.06$
Complexity	$W = 0.95$	$p = 0.054$
Familiarity	$W = 0.74$	$\boldsymbol{p < .001}$
	**Depicted motif-wise (portrait)**	
Liking	$W = 0.96$	$p = 0.79$
Valence	$W = 0.95$	$p = 0.57$
Arousal	$W = 0.93$	$p = 0.20$
Complexity	$W = 0.95$	$p = 0.55$
Familiarity	$W = 0.70$	$\boldsymbol{p < .001}$
	**Depicted motif-wise (landscape)**	
Liking	$W = 0.95$	$p = 0.51$
Valence	$W = 0.95$	$p = 0.44$
Arousal	$W = 0.94$	$p = 0.32$
Complexity	$W = 0.92$	$p = 0.18$
Familiarity	$W = 0.63$	$\boldsymbol{p < .001}$
	**Depicted motif-wise (still-life)**	
Liking	$W = 0.95$	$p = 0.48$
Valence	$W = 0.95$	$p = 0.44$
Arousal	$W = 0.94$	$p = 0.40$
Complexity	$W = 0.96$	$p = 0.74$
Familiarity	$W = 0.81$	$\boldsymbol{p < .001}$
	**Style-wise (representative)**	
Liking	$W = 0.92$	$p = 0.19$
Valence	$W = 0.87$	$\boldsymbol{p = 0.02}$
Arousal	$W = 0.78$	$\boldsymbol{p < .001}$
Complexity	$W = 0.93$	$p = 0.20$
Familiarity	$W = 0.58$	$\boldsymbol{p < .001}$
	**Style-wise (impressionistic)**	
Liking	$W = 0.97$	$p = 0.89$
Valence	$W = 0.96$	$p = 0.77$
Arousal	$W = 0.94$	$p = 0.40$
Complexity	$W = 0.94$	$p = 0.40$
Familiarity	$W = 0.82$	$\boldsymbol{p < .001}$
	**Style-wise (abstract)**	
Liking	$W = 0.91$	$p = 0.35$
Valence	$W = 0.90$	$p = 0.27$
Arousal	$W = 0.94$	$p = 0.40$
Complexity	$W = 0.94$	$p = 0.40$
Familiarity	$W =0.82$	$\boldsymbol{p < .001}$
