# Supplementary figures and images for: Using machine learning to predict judgments on Western visual art along content-representational and formal-perceptual attributes
Source: PLoS One. 2024 Sep 6;19(9):e0304285. doi: 10.1371/journal.pone.0304285 (PMC11379394; doi:10.1371/journal.pone.0304285)

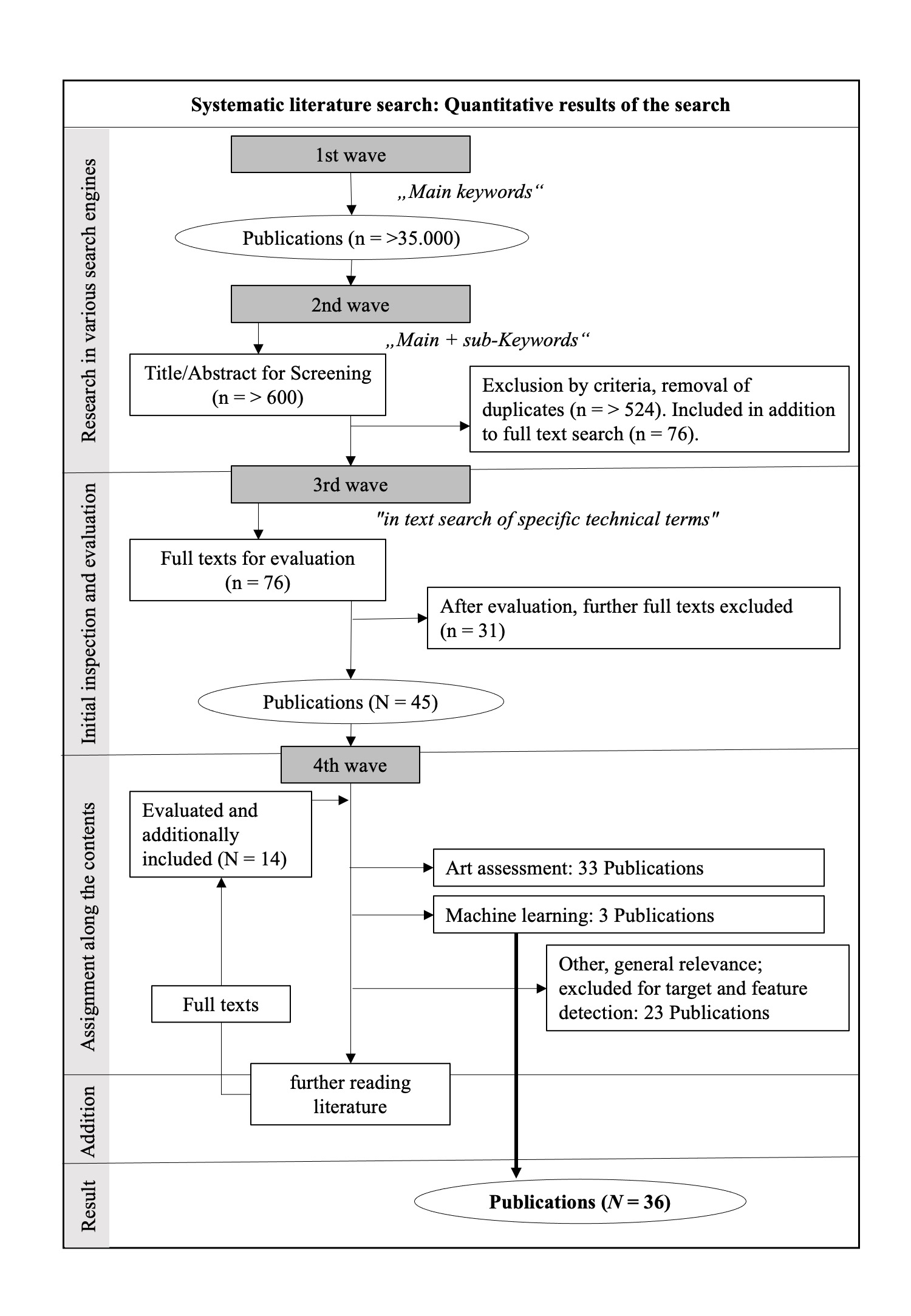

Supplement: S1 Fig — (TIF) [file pone.0304285.s005.tif]

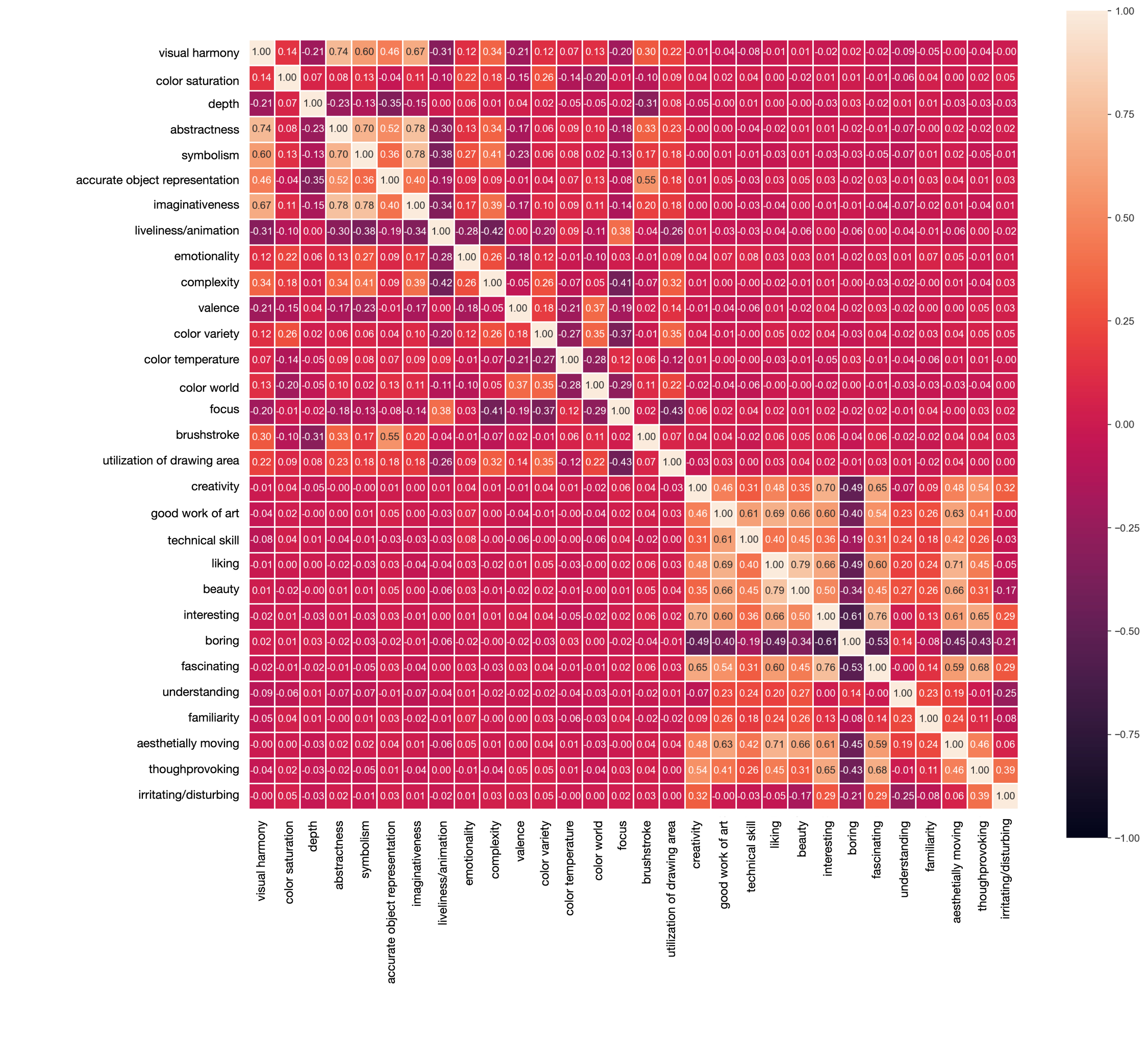

Supplement: S2 Fig — (TIF) [file pone.0304285.s006.tif]
