# Supplementary material for: Using machine learning to predict judgments on Western visual art along content-representational and formal-perceptual attributes
Source: PLoS One. 2024 Sep 6;19(9):e0304285. doi: 10.1371/journal.pone.0304285 (PMC11379394; doi:10.1371/journal.pone.0304285)
Supplement: S1 Appendix — (DOCX) [file pone.0304285.s007.docx]

**S1 APPENDIX**

S1 Appendix Table. Review of art related judgments and attributes. Note the column ‘Description’ summarizes the scales used in reviewed publications; reviewed publications seldom dissociate between felt, expressed, and attributed emotions, art judgments, and art attributes. Column ‘scales included in current study; description of scale adaptions’ specifies which scales we applied in our study; art judgements (see Table 1) are denoted by number; attributes by small letters (see Table 2). See also for judgments selection S1 Appendix Description.

| N**o.** | **Reference (in alphabetical order)** | **Visual art domain** | **Questionnaire or naming of dimensions used (if any)** | **Description** | **Scales included in the current study; descriptions of scale adaptions. See Table 1 and 2 to refer the scale numbering for the current study.** |
| --- | --- | --- | --- | --- | --- |
| 1.0 | **Assessments of art judgments and art attributes** | | | | |
| 1.1 | Bachmann & Vipper  [110] | Paintings |  | 6 semantic differential scales to demonstrate that perception of art evolved:  1. indifferent **-** involved  2. **complex** **-** **simple**  3. **chaotic** **-** **regular**  4. **vague** **-** **precise** | Scales were included as attributes:  1. Indifferent/involved was not included due to unclear meaning  2. complex - simple as (c) complexity  3. chaotic - regular declared as unfitting but semantically covered by (a) visual harmony  4. vague – precise as (j) abstractness, (k) imaginativeness, (l) symbolism (ambiguity), (m) accurate object representation |
| 1.2 | Barron & Welsh [111] [see also, 112,113] | Drawing of figures (non-verbal 86 drawing items) | Barron-Weish Art Scale (BWAS) | Barron-Welsh Art Scale (BWAS) is a subscale contained within Welsh Figure Preference Test (WFPT):  1. **dislike / like**  2. **simplicity / complexity.** | Both included as judgement:  1. dislike / like as (10) liking  2. simplicity / complex as (c) complexity |
| 1.3 | Berlyne [27,28,47,114] | Patterns, line-drawings, animal pictures, polygons, paintings  Study in 1975: 190 possible pairs of exotic and pre-Renaissance paintings | Dimension of aesthetic perception | 12 scales were measured with 7-point Likert type scales along with four factors. Overall, three dimensions of aesthetic perception were detected:  *Uncertainty*  1. **simple, complex**  2. **clear, indefinite**  *Hedonic Value (highest loading factor)*  3. **displeasing, pleasing** 4. **Beautiful, ugly**  5. no pleasure to high pleasure  *Cortical arousal*  6. **weak, powerful** 7. **unbalanced, balanced** 8. drowsy, alert  *Other*  9. **uninteresting, interesting** 10. passive, active  11. no discomfort, high discomfort  12. relaxed, tense | Scales were taken for both art attribute and judgement.  *For art attribute:*  1. simple, complex as (c) complexity  2. clear, indefinite as (l) symbolism and (m) accurate object representation  6. weak, powerful as (o) emotional expressiveness  7. unbalance, balance as (a) visual harmony  *For art judgements:*  4. ugly, beauty as (2) beauty (ugly excluded due to scale number limitation, though considered to be added in future studies)  9. uninteresting, interesting as (7) interesting  Other scales were not used due to three reasons, (1) too vague in meaning, (2) representing emotional judgement response judgements, which we did not investigate in this study, (3) not commonly used in this line of research or critized (see Introduction for discussing the cortical arousal) |
| 1.4 | Biaggio & Supplee [115] | Paintings | Dimension of aesthetic perception (based on Berlyne and Osgood) | The validity of three dimension of art perception (reported in 1.3) was tested for two groups, i.e., art and non-art knowledgeable participants, using the same scales. Additional scales used were orderly/disorderly.  **Hedonic interest** (highest factor loadings art: 1, 2, 12, 6, 7, 8; non-art: 1, 2, 12), **Arousal** (art: 9, 10,11 non-art: 9, 10, 6, 7, 8), and **Uncertainty** (art/non-art: 3, 4, 5) | See for integrated scales 1.3 |
| 1.5 | Chatterjee, et al. [18] | Paintings | Art experience questionnaire, | 8 paintings assessed on two dimensional concepts (formal and conceptual attributes); participants rated on a 5-point Likert scale.  ***Formal-perceptual attributes***  **1. balance (visual harmony or visual rightness)**  **2. color temperature (warm, cold)**  **3. color saturation (calm, vibrant)**  **4. stroke style (controlled, loose)**  **5. depth with flat (flat, deep) or depth (sense of three dimensions)**  **6. complexity (simple, complex)**  ***Content-representational attributes***  **1. objective accuracy (degree of depictive realism)**  **2. abstractness (abstract, concrete)**  **3. realism (realistic, fantastic)**  **4. animacy (more or less sense of the objects being alive)**  **5. symbolism (literal, symbolic)**  **6. emotional expressivity (more or less emotional expressivity).** | All scales were included. A general adaption was made for the content-representational attributes. We also used word pairs with opposite poles (see Table 2 for all attributes in the present study). |
| 1.6 | Che et al. [69] | Scene perception |  | Assessment of cultural differences basing aesthetic preferences on a common set of formal features:  1. symmetry  **2. complexity**  3. proportion  4. contour  **5. brightness**  6. contrast | Scales of formal features were included:  *For art attributes:*  1. simple, complex as (c) complexity |
| 1.7 | Cupchik & Berlyne, [5] | Paintings | Collative properties in visual stimuli | The three factors detected by Berlyne and Ogilvie (1974) and Osgood et al. (1957) were evaluated with 7-point Likert type scales:  *Uncertainty factor* (Osgoods activation dimension):  **1. simple, complex**  **2. disorderly, orderly**    *Hedonic tone factor* (Osgood's evaluation dimension):  **3. displeasing, pleasing**  *Arousal factor* (Osgoods potency dimension):  4. drowsy, alert  5. relaxed, tense | Scales were taken for both art attribute and judgement.  *For art attributes:*  1. simple, complex as (c) complexity  2. disorderly, orderly was semantically  *For art judgement:*  3. displeasing, pleasing as (13) liking  drowsy, alert appeared as more emotional factors and were not included. This also accounts for relaxed, tense. |
| 1.8 | Eysenck [116,117] | Paintings and other visual artifacts (statues, book-bindings, etc.)  Paintings and photographs |  | 18 sets of pictures including portraits, drawings, japanese paintings, curves and other visual artifacts were ordered along **liking**  217 pictures were used to rank the pictures in each set ranked after a **liking** scale, using a scheme of grouping which closely approximated to the normal distribution curve. | liking (for Eysenck was the amount of liking the true aesthetic value) as (13) liking. Aesthetic aspects were represented by (1) aesthetically moving and (2) beauty. |
| 1.9 | Haanstra et al. [108] | Drawings from different people (children, artists, other grown-ups) |  | Drawings were evaluated by judges with different art expertise along with 5-point Likert type scales:  1. **interestingness**  **2. pleasingness**  **3. overall quality** | Scales were taken for both art judgements.  1. Interesting as (7) interestingness  2. Pleasingness as (13) liking  3. Overall quality we integrated along qualitative aspects as (3) good work of art, (4) creativity, and (5) technical skill. |
| 1.10 | Hager et al. [118] | Painting | Art Reception Survey (ARS) | 29 items with 5-point Likert type scale (1 = *completely disagree*, 5 = *completely agree*) consisting 6 factors (4–5 items each):  1. **cognitive stimulation** (e.g., **makes me curious, is thought-provoking, exciting to think about, is fun to deal with**)  2. negative emotionality (e.g., makes me feel afraid, makes me sad, makes me feel troubled, makes me feel lonesome)  3. expertise (e.g., can relate painting to its art historical context, can relate painting to a particular artist, know this painting, have an idea what artist is trying to convey)  4. self-reference (makes me think about my own life history, can associate painting with personal biography, personal memories linked to painting, painting mirrors own personal emotional state)  5. **artistic quality (e.g., is unique, features a high level of creativity, composition is of high quality, artist’s manner of painting is fascinating)**  **6. positive attraction (e.g., is pleasant, is beautiful, thrills me, feel inspired by this painting)** | Scales were taken for both art attribute and judgement.  *For art attribute:*  2. Negative emotionality as (o) emotional expressiveness.  *For art judgements:*  1. cognitive stimulation as III. epistemic aspects with (6) fascinating (intellectual), (7) interesting, and (8) though provoking.  5. Artistic quality was covered by our II. qualitative aspects with (3) good work of art, (4) creativity, and (5) technical skill.  6. Positive attraction was separated in I. aesthetic aspects and II. preference aspects.  Expertise is not an art-attribute but a person-related factor, which was collected separately from the ratings, and self-reference was not focus of our study. |
| 1.11 | Hagtvedt et al. [119] | Visual art (paintings) | Measurements of the affective and cognitive components involved in the perception of visual art | 35 items, 9 factors (3–5 items each): *Emotion factors*  1. **negative emotion**, high arousal (unease, anxiety, uncertainty, disquiet)  2. **negative emotion,** low arousal (sadness, despair, gloom, loneliness)  3. positive emotion, high arousal (excitement, enthusiasm, thrill)  4. positive emotion, low arousal (happiness, joy, gladness, serenity); Cognitive 19factors  5. **curiosity** (interesting, arousing curiosity, fascinating, intellectually stimulating)  6. **aesthetic** (aesthetically, attractive, beautiful, appealing)  7. **creativity** (original, distinct, creative, inventive)  8. **skill** (workmanship, well crafted, skillfully made); Evaluation index: 9. **evaluation** (good, positive, favorable, pleasing, like) | Scales were taken for both art attribute and judgement.  *For art attribute:*  1. to 4. Was included with the general attribute (p) valence and emotionality as (o) emotional expressiveness.  5. curiosity as III. epistemic aspects: (6) fascinating (intellectual), (7) interesting, and (8) though provoking.  6. aesthetic as I. aesthetic aspects: (1) aesthetically moving.  7. creativity as (4) creativity.  8. skill as (5) technical ability.  9. evaluation as (13) liking, and as attribute (p) valence. |
| 1.12 | Hawley-Dolan et al. [120] | Visual artworks |  | **1. Liking** and **2.** **good work of art** with 7-point Likert type scale (liking: 1 = not at all, 7 = very much, good work of art: 1 = no, absolutely not, 7 = yes, absolutely) including contextual changes (stories telling art was made either by accident or intentionally). Note that morality judgements for different scenarios were also included in the experimental design. But, this judgement was irrelevant from the judgement towards artworks, and thereby, was over the scope of the present study. Thereby, it was not considered for the scale inclusion. | Scales were taken for art judgement.  1. liking as (13) liking  2. good work of art as (3) good work of art |
| 1.13 | Kim et al. [121] | Paintings |  | 40 paintings were randomly selected from the set of 100 paintings were rated along **attractiveness**. | Attractiveness did not appear to be appropriate for our study. |
| 1.14 | Leder et al. [34]  see also for update Leder & Nadal [1] |  |  | Model of art and aesthetic appreciation. Stages reported:  1. pre-classification (interest)  2. perceptual analysis (**fluency**, **complexity**, contrast, **symmetry**, order, grouping)  3. memory integration (**familiarity**, fluency, prototypicality)  4. explicit classification (content, style)  5. cognitive mastering, evaluation (**understanding**, ambiguity, satisfaction, **pleasure**) | Though this study is rather conceptual, some stages could be corresponding to specific attributes/judgements.  *For art attribute:*  For stage 2. perceptual analysis, (a) visual harmony, (c) complexity, (j) abstractness (which is also a style wise attribute), (l) symbolism (ambiguity), (m) accurate object representation could be included.  *For art judgements:*  3. memory integration (familiarity) as (11)  5. cognitive mastering (understanding) as (12) understanding, and (pleasure) as (13) liking. |
| 1.15 | Leder, H., Gerger, G., Dressler, S. G., & Schabmann, A. (2012) | 24 paintings (Classic, modern, abstract) |  | Following scales were measured for 24 paintings with 7-point Likert type scale (1 = not at all/negative, 7 = very much/very positive)  **1. liking**  2. elicited emotions  **3. arousal**  **4. comprehension** | Scales were taken for art attributes and judgement.  *For art attribute:*  1. liking as (13) liking  4. comprehension as (12) understanding  *For art judgements:*  3. arousal semantically as (o) emotional expressiveness  Elicited emotional types are beyond the scope of the present study. Emotional aspects are only covered by (o) emotional expressiveness and (p) valence. |
| 1.16 | Leder et al. [36] | Art portraits  #Study 1 with 420 portraits  #Study 2 with 96 portraits chosen from the stimuli from Study 1  #Study 3 with the identical stimulus set of Study 2 |  | Following scales were measured for 24 paintings with 9-point Likert type scale (1 = lowest/negative, 9 = highest/very positive)  **1. likability of the depicted person**  2. evaluation of the portrait’s style 3. arousal (not in Study 2 and 3)  **4. interestingness** (not in Study 2 and 3)  **5. emotional valence**  **6. aesthetic liking**  **7. familiarity**  Note that, in Study 3, two additional scales were added to assess how much the the overall (aesthetic) liking was affected by likability and style appreciation:  A 5-point Likert scale from -2, very negative influence to +2, very positive influence, and 0, no influence was assigned. Neverhtless, as this scales were specifically added for their research questions, they were not concerned for the scale inclusion of the present study. | Scales were taken for art attributes and judgement.  *For art attribute:*  1. likability as (13) liking. Note that we did not differ between likability of a potential depicted person or item in the image.  4. interestingness as (7) interesting  7. familiarity as (11) familiarity  *For art judgements:*  5. emotional valence as (p) valence  We did not include arousal, as discussed in the introduction. |
| 1.17 | Marin et al. [32] | 96 affective environmental scenes [taken from the International Affective Picture System (IAPS), Lang et al., 2005], cartoons, and 96 representational paintings |  | Following scales were measured for stimuli with 7-point Likert type scale (1 = lowest/negative, 7 = highest/very positive). Ratings were taken for hedonic tone based on Berlyne:  **(1) beauty**  **(2) pleasantness**  **(3) liking**  (4) arousal  **(5) complexity**  **(6) familiarity** | Scales were taken for art attributes and judgement.  *For art attributes:*  (5) complexity as (c) complexity  *For art judgements:*  (1) beauty as (2) beauty  (2) pleasantness and (3) liking as (13) liking  (6) familiarity as (11) familiarity  (c) Complexity in our study was included as attribute.  We did not include arousal, as discussed in the introduction. |
| 1.18 | Martindale [30] | Random polygons, shapes, drawing and paintings |  | Semantic differentials used as scales for art stimuli:  **complex-simple, meaningless-meaningful, like-dislike, orderly-disorderly, nonphotographic-photographic, natural-unnatural, and static-dynamic** Note the seven studies included different scales, specifically:  Study 1: liking  -10 dislike a lot + 10 like a lot  Study 2: liking  1 like a lot 7 dislike a lot  Study 3:  7-point bipolar scales  like-dislike  meaningless-meaningfull  orderly-disorderly  complex-simple  Study 4: liking  -3 (dislike a lot) 0 (neutral) +3 (like a lot)  Study 5:  7-point bipolar scales  Complex-simple  Meaningless-meaningfull  Like-dislike  orderly-disorderly  nonphotographic-photographic  natural-unnatural  static-dynamic  Study 6:  7-point bipolar scales  Like-dislike  Interesting-uninteresting  Simple-complex  orderly-disorderly  continuous-discrete  indefinite-clear  shapes unimportant-shapes important  weak-strong  static-dynamic  meaningless-meaningful  composition or arrangement unimportant-important  not photographic-photographic  unnatural-natural  not representative of reality – representative or reality  objective idea – inner feeling  surface texuture unimportant – surface texture important  Study 7: same as study 6  Additionally, ‘how much they know about art in general’ | Study replicating results from Berlyne, which could not be supported. Results showed association between liking as preference with meaning and complexity.  We included as judgments: (6) Fascinating (intellectual), (8) though provoking as potentiall covering meaningful; we further included (13) liking and as attributes (c) complexity, (a) visual harmony as orderly item, (m) Accurate object representation for photographic depiction, (k) imaginativeness for un/natural, and (o) animacy for static, dynamic |
| 1.19 | Mayer & Landwehr [62] | Abstract artworks, landscape photographs |  | Link between visual inputs and evaluative response was assessed via following scales with horizontal slider (with 100steps from not at all to very much). **Liking** and four algorithmic measures: **visual simplicity, visual symmetry, visual contrast,** and **visual self-similarity**   Note, the three studies used different measures/scales, specifically:  Study 1  Aesthetic liking  Subjective fluency experience  Study 2  Aesthetic liking  Subjective fluency experience  Symmetry  Simplicity  Contrast  Self-similarity  Study 3 seems to only have behavioral metrics (number of downloaded pictures etc) | Included as attribtues: visual simplicity as (c) complexity, visual symmetry as (a) visual harmony, visual contrast partially covered as (g) color world, and visual self-similarity partially semantically covered by (a) visual harmony. Note we did not include these measures as objective ones. |
| 1.20 | Miller & Hübner [122] | 24 pictures of diverse paintings |  | Dimensions of the study were  (1) **positive attraction** dimension (“this painting is beautiful”)  (2) **cognitive stimulation** dimension (“this painting is thought-provoking”)  (3) **emotionality dimension** (“This painting causes emotions”)  Note that the response was recorded via a continuous rating scale (from 1 = not at all to 101 = very much for the Positive Attraction and Cognitive Stimulation scales, and -50 = negative to 50 = positive on the Emotionally scales). | Scales were taken for art attributes and judgement.  *For art attributes:*  (3) emotionality dimension as (o) emotional expressiveness    *For art judgements:*  (1) positive attraction as I. aesthetic aspects and VI. Preference aspects  (2) cognitive stimulation as (8) though-provoking. |
| 1.21 | O’Hare and Gordon [123] | Paintings |  | Use of same semantic differentials by Berlyne (1973); resulting in most important factor pairs: **realistic-unrealistic, clear-indefinite, and symmetrical-asymmetrical dimensions to the preference of paintings** | Attributes included for our study are (k) imaginativeness, symbolism (ambiguity) with poles distinct/clear-symbolic (more room for interpretation, ambiguous) instead of indefinite, symmetrical item was included as (a) visual harmony. |
| 1.22 | Ortner [124]  Panagl[125] | Modern art pictures | Wordlist for the assessment of emotions in response to modern art | 19 items, 3 factors (6–7 items each): (1) **liking/interest** (e.g., pleased, sympathetic, fascinated, ruminative), (2) **negative affect** (e.g., uneasy, threatened, pessimistic, stressed), (3) **repulsion/aggression** (e.g., angry, aggressive, repulsive, disgusting) | Both, (13) liking, and (7) interesting was included. We did not focus during this study on affective responses, though we included (o) emotional expressiveness, and (p) valence. |
| 1.23 | Palmer et al. [65] and Palmer et al. [63] | Aesthetic viewpoint |  | Theoretical discussion:  (1) **preference ratings**  **(2) color** (hue, saturation, lightness, hue x lightness interactions) including harmony (color combination)  (3) **spatial structure** (golden ratio, complexity and symmetry, contour curvature, categorical prototypes)  (4) **spatial composition** (structure of the rectangular frame, balance and centers, compositional biases in pictures of meaningful objects, higher level spatial composition: effects of meaning, titles, and context)  (5) **aesthetic response** (mere exposure, arousal dynamics, prototype theory, fluency theory) | Though this study is rather theoretical, some factors listed in this literature could be corresponding to specific attributes/judgements.  *For art attributes:*  (2) color as (d) color saturation, (e) color variety, (f) color temperature, (g) color world  (3) spatial structure as (c) complexity  (4) spatial composition as (i) utilization of drawing, (q) focus  *For art judgements:*  (5) aesthetic response semantically as (j) abstractness, (l) symbolism |
| 1.24 | Pelowski, et al. [35] |  |  | Major update of model of art appreciation by Leder et al. (2004). For stage viewpoint see 1.14. Further discussion of factors such as **(1) being moved**  (2) chills  (3) anxiety  (4) anger  (5) confusion  (6) epiphany  **(7) harmony** | Though this study is rather theoretical, some factors listed in this literature could be corresponding to specific attributes/judgements.  *For art attributes:*  (7) harmony as (a) harmony - but not as elicited emotion.  *For art judgements:*  (1) being moved as (1) aesthetically moving  Others were excluded Please my see suggestion here as the other items are listed specifically as elicited emotions, and thereby, beyond the scope of the present study.. |
| 1.25 | Pelowski et al., [126] | Art productions and realistic copyings |  | Following scales were measured for stimuli with 7-point Likert type scale (1 = extremely poor, 7 = extremely good):  (1) **quality as an artwo**rk  (2) **creativity**  (3) **realism**  (4) **liking** (personal liking) | Scales were taken for art attributes and judgement.  *For art attributes:*  (3) realism as (k) imaginativeness, also semantically in (l) symbolism  *For art judgements:*  (1) quality as an artwork as (3) good work of art, and (5) technical skill  (2) creativity as (4) creativity  (4) liking as (13) liking |
| 1.26 | Schindler et al. [59] | Different domains: arts (literature, music, visual art, film, etc.) and aesthetically appealing sights and sounds beyond the traditional arts (advertising, consumer products, natural beauty, etc.) | Aesthetic Emotions Scale (AESTHEMOS) | 21 sub-scales with two items each (i.e., 42 items in total) to measure specific aesthetic emotions of prototypical aesthetic emotions are: **(1) feeling of beauty, (2) liking/attraction,** (3) captivation, **(4) being moved,** (5) awe, (6) enchantment/wonder, and (7) nostalgia/longing; the pleasing emotions are: (8) joy, (9) humor, (10) vitality/arousal, (11) energy, and (12) relaxation; the epistemic emotions are: (13) surprise, (14) interest, (15) intellectual challenge, and (16) insight; the negative emotions are: (17) feeling of ugliness, (18) disliking/displeasure, **(19) boredom,** (20) confusion, (21) anger, (22) uneasiness/fear, and (23) sadness; and the single self-forgetful emotion is: (24) flow/absorption. | Focus of their review was covering aesthetic emotions. We also included feeling of (2) beauty, and (1) aesthetically moving as I. aesthetic aspects. (19) boredom was included as judgment (11) boring. (2) Liking/attraction was also covered by our VI. Preference rating (13) liking.  Our focus was however not fully on emotional and self-response aspects and further items were not covered in our study. |
| 1.27 | Sidhu et al. [13] | 240 abstract and 240 representational paintings |  | Prediction of beauty and liking ratings by subjective/objective ratings for the paintings.  (1) **beauty** and (2) **liking** were measured via 9-point Likert type scale (1 = ugly/dislike, 5 = neither ugly nor beautiful / neither dislike nor like, 9 = beautiful/like).  Four subjective predictors also with 9-point Likert type scale in the same manner:  (3) **meaningfulness**  (4) **complexity**  (5) **emotionality**  (6) **color warmth**  Objective measures:  **(7) hue**  (8) **saturation**  (9) **value** (henceforth brightness; HSV) model of color. | Scales were taken for art attributes and judgement.  *For art attributes:*  (4) complexity as (c) complexity  (5) emotionality as (o) emotional expressiveness  Note that, in Sidhu et al. (2018), the objective measures were retrieved by the pixel information in each image, thus as objective information in the pictures. However, in the present study, such dimensions were also subjectively asked to the participants.  (7) hue as (f) color temperature, and (g) color world  (8) saturation as (d) color saturation  (9) value as (g) color world  *For art judgements:*  (1) beauty as (2) beauty  (2) liking as (13) liking |
| 1.28 | Silvia [127,128] | Visual art |  | Review based on Berlyne’s research how appraisal theory of emotions informs the study of aesthetics.  Study used multilevel modeling assessed the within-person effects of appraisals on interest. | Elicited emotions were not included in the study. However, as judgment we also had (7) interesting as target. |
| 1.29 | Specker et al. [129] | Paintings | Aesthetic effects network (AEN) | 14 differential bipolar scales:  **(1) negative–positive**  **(2) passive–active**  (3) lively–still  (4) happy–sad  (5) aggressive–peaceful  (6) soft–hard  (7) warm–cold  (8) heavy–light  (9) smooth–rough  (10) bodily–spiritual  (11) masculine–feminine  (12) intrusive–cautious  **(13) dislike-like**  **(14) uninteresting-interesting** | Scales were taken for art attributes and judgement.  *For art attributes:*  (1) negative-positive as (p) valence  (2) passive-active as (n) liveliness/animation  *For art judgements:*  (13) dislike-like as (13) liking  (14) uninteresting-interesting as (7) interesting  We did not include dimensions of happy-sad, aggressive-peaceful, or soft-hard, etc. as they did not appear to be artwork specific attributes (see also Chatterjee’s et al. (2010) approach) |
| 1.30 | Tröndle et al. [130] | Fine art museum exhibition | Questionnaire on subjective aesthetic experiences | Self-report measure covering evoked emotions, aesthetic evaluations, and general appraisal of an artwork with 5-point Likert type scale; questionnaire is part of an integrative methodology assessing locomotion and physiological data;  Scales (19 items, 5 factors):  (1) **aesthetic quality** (e.g., pleasing, beautiful, emotionally moving)  (2) **surprise/humor** (e.g., surprising, makes one laugh, makes one think)  (3) **negative emotion** (e.g., work conveys sadness, fear, anger)  (4) dominance (e.g., work experienced as dominant, stimulating)  (5) curative quality (e.g., work is well staged and hung, suitable in the context of other artworks) | Scales were taken for art attribute and judgement.  *For art attributes:*  (3) negative emotion as (p) valence  *For art judgements:*  (1) aesthetic quality as (1) aesthetically moving  (2) surprise/humor as (8) thought provoking  Dominance, and curative quality were not included due to limited number of items we could include in the study and unsuitable structure. |
| 1.31 | Tschacher et al. [131] | Artworks in real-life museum |  | Museum study with physiological measures and aesthetic assessment including emotion category from Ekman and Friesen (1971), emotion dimensions from, RusseIl and Mehrabian (1977), and negative emotions from Silvia (2009).  For Aesthetic assessment, 19 items were used yielded five factors:  (1) **aesthetic quality** (the work is rated as pleasing; beautiful; well done with respect to technique, composition, and content)  (2) **surprise/humor** (the work is considered as surprising; makes one laugh)  (3) **negative emotion** (the work conveys sadness, fear, anger)  (4) dominance (the work is experienced as dominant, stimulating)  (4) curative quality (the work is well staged and hung, suitable in the context of other artworks) | Scales were taken for art attribute and judgement.  *For art attributes:*  (3) negative emotion as (p) valence  *For art judgements:*  (1) aesthetic quality as (1) aesthetically moving  (2) surprise/humor as (8) thought provoking  Dominance, and curative quality were not included due to limited number of items we could include in the study and unsuitable structure. |
| 1.32 | van Paasschen, et al. [132] | Paintings and portraits (100 abstract artworks and 50 portraits that were all part of the exhibition ‘La Magnifica Ossessione’ held at the Mart) |  | Following scales were measured for stimuli with a 7-point Likert type scale on:  **(1) valence**  (2) arousal  **(3) beauty**  **(4) liking.** | Scales were taken for art attribute and judgement.  *For art attributes:*  (1) valence as (p) valence  *For art judgements:*  (3) beauty as (2) beauty  (4) liking as (13) liking  We did not include arousal as separate judgement or attribute (see introduction for this reason). |
| 1.33 | Wanzer et al. [133] |  | Aesthetic Experience Questionnaire (AEQ) | The study aim was to validate the questionnaires (AEQ). Each item was evaluated with a 7-point Likert type scale (1 = strongly disagree, 7 = strongly agree).  (1) Emotional (e.g., I experience a wide range of emotions.)  Cultural (e.g., I compare the past culture of the art with present-day culture.)  (3) Perceptual (e.g., The composition of a work of art is important to me.)  (4) Understanding (e.g., I try to understand the work completely.). (5) Flow – Proximal Conditions (e.g., I have a clear idea of what to look for when viewing the work of art.)  (6) Flow – Experience (e.g., I lose track of time when I view the work of art.) | None of the items were not precisely adapted in the present study.  Even though this study covers interesting items for self-perception, the items were developed to capture specifically the aesthetic experiences proposed by Csikszentmihalyi and Robinson (1990). As such, the items do not seem like to present an appropriate input for our study. |
| 2.0 | **Art and machine learning studies** | | | | |
| 2.1 | Birkhoff [46] |  | Aesthetic Measure | Formula suggested to quantify the aesthetic measure as *M* = 0 / C or as function of this ratio M = f(OC). O = order, and C = complexity. | Not a machine learning approach itself but major paper in this line of research. The aesthetic measure we included as judgments primarily with (13) liking but also with (1) aesthetically moving, (2) beauty.  Order or balance we added as (a) visual harmony (balance) and (c) as complexity |
| 2.2 | Li & Chen [48] | Paintings |  | Visual quality assessment using machine learning: Color, composition, brightness separated for global features (over all the pixels of the image) and local features (segments of pixels) | Objective measures were not taken at this present stage. However, we included 4 color attributes, which were color (d) saturation, (e) variety, (f) temperature, and (g) color world as subjective ratings. |
| 2.3 | Li et al. [49] | Evolutionary art |  | Machine learning approach investigating preference in evolutionary art looking at the following art features:  (1) color moments  (2) lightness feature  (3) texture feature  (4) image complexity Image order  (5) MC metric, the image complexity and processing complexity ratio | We did not include objective measure in our current study.  Nevertheless, color aspects and complexity aspects were subjectively asked to the participants in art attributes.  (1) color moments as (d) color saturation, (e) color variety, (f) color temperature, and (g) color world  (4) image complexity as (c) complexity |
| 2.4 | Iigaya et al. [50] | Paintings |  | Machine learning approach predicting **liking** measure via 4-point Likert type scale (7 participants in a lab all 1001 paintings, 1,359 participated online study rated approximately 60 paintings) via two features:  *high-level features* (subjectively measured via 13 extra participants as in the same manner as AAA (see 1.5).  (1) **concreteness** (abstract-concrete)  (2) **dynamics** (dynamic-still)  (3) **temperature** (hot-cold)  (4) **valence** (positive-negative)  *low-level feature*  (5) hue contrast  (6) brightness contrast  (7) blurring effect  (8) vertical center of largest segment  (9) saturation of 2nd largest segment  (10) blurring contrast between segments  (11) size of largest segment  (12) width-height ratio  (13) presence of a person | Liking as (13) liking  Scales in high-level features were taken for art attribute and judgement.  (1) concreteness as (k) imaginativeness and (l) symbolism, (m) accurate object  (2) dynamics as (n) liveliness/animation  (3) temperature as (f) color temperature  (4) valence as (p) valence  Objective measures were not included yet at this stage of this research. |
| 3.0 | **Additional papers, which were not the focus of our study but present other interesting and relevant scales or approaches for studying art and aesthetic experiences including major review papers** | | | | |
| 3.1 | Barhami-Ehsan et al. [134]  See also Afhami & Mohamadi-Zarghan [135] | Judgmental declarative sentences about various arts | Aesthetic Judgment Style Scale (AJSS): three subscales of Sternberg’s additional: thinking Styles Inventory (TSI), and cognitive style scale (CSS) | 4 sub-scales to assess 4 major aesthetic judgment styles each 8 items 6-point Likert type scale ranging from ‘completely agree’ to ‘completely disagree’:  (1) **Concrete aesthetic judgment style** includes judgments which describe artworks by their apparent and superficial qualities. In this style of judgment, one does not display deeper interpretations of the work.  **(2) Analytical aesthetic judgment style** involves making inferences considering artistic guidelines. People with this style of aesthetic judgment make an effort to analyze the artwork and make logical statements about it.  **(3) Symbolist aesthetic judgment style** consists of making judgmental comments apart from concrete or practical ones. References in this style are not directly related to specific instances. Moreover, judgments do not have narrative content or pictorial representations. (4) **Emotional aesthetic judgment style** forms when affective and emotional statements are greatly used in an aesthetic judgment. One has an emotional bond with the artwork and considers it very close. This judgment style may be considered as an advanced style which is more frequently seen in experienced artists or art critics. | Judgements investigations in the study has focus on personality traits. Accordingly, such scales were not included yet at this stage of this research with potential interests for the future research. |
| 3.2 | Corradi, et al. [136] | Visual pattern #Study 1 has 66 forms used in VAST  #Study 2 has 24 stimuli selected from Study 1  The stimuli were categorized into curved-counters, sharp-angled, symmetry-asymmetry, simple-complex, balance-unbalance | Conception of aesthetic sensitivity: Visual Aesthetic Sensitivity Test (VAST) | Liking was rated for each stimulus with 7-point Likert type scale (1 = I don’t like it at all, 7 = I like it a lot) | Liking as (13) liking  Some categorizations for stimuli in this study was addressed in our art attribute:  Complexity as (c) complexity  Balance as (a) visual harmony.  curve-contour and symmetry-asymmetry were not included in the scales, as these were specifically chosen as attribute for visual patterns and not artworks. |
| 3.3 | Chamorro-Premuzic & Furnham, [137] | Paintings with different styles and figures | NEO-PI-R (Costa & McCrae, 1992)  the Wonderlic Personnel Test (Wonderlic, 1992) the Raven Progressive Matrices (Raven, Raven & Court, 1984)  Meier art judgement test (Meier, 1940) | Eight weeks after answering all questionnaires, one-page questionnaire on art interests, art activities and art preferences were asked. 100 slides with (at least) a pair of figures (design) were presented to the participants, and they answered if there were ‘better’ (correct or real) or not. | As a general results, personality and intelligence explained 25% of the variances in art judgement scores. Nevertheless, as there was no specific evaluation(s) made towards stimuli, thus, there were no further implementations for our scales. |
| 3.4 | Csikszentmihalyi & Robinson [138] |  |  | Specific qualitative and quantitative assessment of art professionals covering perceptual, intellectual, emotional, and knowledge dimensions along interviews and expertise measures (occupation, education, etc.) | Interesting focus on art experts, thus, there were no further implementations for our scales. |
| 3.5 | Cupchik [139,140]  see also Cupchik & Hilscher [141] and Cupchik et al. [15] | Other aesthetic object, design, and also visual art |  | Review and investigations of elicited emotions | Non-relevant factors for our study, though fundamental research for studying emotional response, also brain investigations |
| 3.6 | Ekárt et al, [142] | Evolutionary art |  | 500 sessions of evolutionary art systems measured on **liking** | We included liking as (13) liking; however, as this was computer-based art but still judged by human participants, no machine learning approach was taken for analysis. |
| 3.7 | Eysenck, [60] | Color paper sheets |  | Investigation of color and saturation. | Not focus of our study, but important for beginnings of investigation of color perception and preference. |
| 3.8 | Israeli [143] | Paintings | List of emotion adjectives | 23 scales comprising 1–5 emotion adjectives to describe emotional reactions to painting reproductions: (1) calmness (e.g., peace, serenity)  (2) depression (e.g., sadness, melancholy)  (3) solitude  (4) fatigue  (5) excitement (e.g., anxiety, restlessness)  (6) frenzy  (7) tumult (e.g., agitation, confusion)  (8) surprise  (9) wanderlust (e.g., adventurous, yearning)  (10) wonder (e.g., curiosity)  (11) delight (e.g., happiness, pleasant)  (12) exaltation (e.g., exhilaration, ecstasy)  (13) sprightly (e.g., vivacious, cheerful)  (14) romantic  (15) awe (e.g., sublimity, majestic), (16) apathy (e.g., laziness, stupor), (17) sympathy  (18) unpleasantness  (19) love  (20) fear  (21) anger  (22) hate  (23) admiration | Our focus was not to elicited emotional aspects at this stage of research. (o) emotional expressiveness was included as an artwork specific attribute, not the personal affective response by the viewers.  Nevertheless, this study nicely shows which emotional aspects can be included as scales. |
| 3.9 | Jonauskaite et al. [61] |  |  | Machine learning approach studying color-emotion association between and their cultural differences | Machine learning approach, though no visual-art investigation. Focus on color attributes. |
| 3.10 | Lundy et al. [144] |  | Desire for Aesthetics Scale (DFAS) | 36 scales evaluating the desire for aesthetics in everyday life through various lifestyles. | Though not focus of our study, it contains questions connecting aesthetic judgements and lifestyles. |
| 3.11 | Marković [145], see also further research [146] | Paintings | Descriptors of the aesthetic experience and emotional content of paintings | 22 items with 7-point Likert type scale, along two factor groups:  (1) affective tone (14 items: lovely, charming, cheerful, scary [reversed], disgusting, hateful tragic, sad, pitiful, disappointing, angry)  (2) aesthetic experience (8 items: exceptional, profound, unique, awing, delightful, eternal, unspeakable) | Our focus was not to cover elicited personal emotional response aspects at this stage of research. (o) emotional expressiveness was included as an artwork specific attribute, not the personal affective response by the viewers. |
| 3.12 | Martindale [31] |  |  | Review paper of the psychological study of aesthetics, creativity, and the arts | As it has theoretical focus, no further implementations for our scales. |
| 3.13 | Menninghaus et al. [147] | Recall (imagining) of own-life experiences, media-representedreal events, or (fictional) artworks | GAQ (Geneva Appraisal Questionnaire) | Investigation of being moved, being stirred, and being touched along several imagined recalls of events and affective questionnaires. | Although we included (a) aesthetically moving as art judgment, we did not focus on elicited being moved in our study. |
| 3.14 | Murray [148]  Lu et al. [149,150] | Images | AVA, Rapid visual aesthetics using deep learning (RAPID) | Analysis of large-scale database for aesthetic visual analysis | Machine learning approaches for image classification. No visual art though extensive approaches investigating objective image attribute elements. |
| 3.15 | Nadal & Ureña [29] |  |  | Review of empirical aesthetics. | Extensive review of research in empirical aesthetics from Fechner to Berlyne, including discussion of used scales. |
| 3.16 | Osgood [72,151] see also Osgood et al. [152] and Adams & Osgood [105] |  | EPA model | Semantic differential technique along many different factors also with between-culture comparison  **Evaluation**, **Potency**, and **Activity** model (EPA model; see Wellek, 1929 for a similar model focused on cross-modal correspondences of aesthetic effects based on historical research) | Extensive investigation of validity and reliability of semantic differential technique. As his focus was not on non-art stimuli it was not directly relevant for our study regarding. |
| 3.17 | Pelowski et al. [153]  See also Pelowski & Akiba [154] | Mark Rothko paintings |  | Investigation of ‘feeling like crying’ initiated by an art and aesthetic experience. Elicited emotions measured were:  (1) cognitive mastery/discrepancy: anxiety, confusion, tension, surprise  (2) secondary control: need to leave  (3) self-aware reflection: self-awareness, felt being watched, changed my mind, examined motives for viewing, felt the paintings watching me, aware of my actions  (4) aesthetic phase/insight: epiphany, understood artist intention, sadness, happiness, relief, time | Thorough review and study of intense emotional aesthetic experiences. Measures focused on elicited emotions, which was not focus on our study |
| 3.18 | Pelowski, et al. [155] |  |  | Major review of psychological art models. | Extensive review of art experience models. |
| 3.19 | Rowold [156] | Visual art in general including paintings and sculptures | Survey for the Assessment of Aesthetic Perception (SAAP) | 3 factors:  (1) **cognition (**5 items: have to think about artwork, exciting, content of artwork occupies my mind, provides me with new information)  (2) **emotion** (4 items: relaxed, feel good, feel fresh, feel colorful)  (3) **self- congruency**: (7 items: reconsidering my personal life, remember my life history, discover new aspects of myself, artwork has something to do with myself) | Different methodological structure of items, which were not suitable for our study. |
| 3.20 | Silvia et al. [100,157] | Aesthetic Experiences Scale/Unusual Aesthetic Emotions Scale | Aesthetic Experiences Scale/Unusual Aesthetic Emotions Scale | 10 items, 3 factors (2–5 items each): (1) **chills** (feel chills down your spine, feel like your hair is standing on end, get goose bumps)  (2) **feeling touched** (feel touched, feel like crying)  (3) **absorption** (e.g., feel absorbed and immersed, completely lose track of time, feel like you’re somewhere else) | We did not focus on chills, feeling touched, or absorption in our study. However, we included (1) aesthetically moving as aesthetic experience. |
| 3.21 | Smith & Smith [158] [see also, 159] | Visual art in museum | Aesthetic fluency scale | 10 items investigating knowledge of artists and ideas in art history (Mary Cassatt, Isamu Noguchi, John Singer Sargent, Alessandro Boticelli, Gian Lorenzo Bernini, Fauvism, Egyptian Funerary Stelae, Impressionism, Chinese Scrolls, Abstract Expressionism) | Art knowledge, art understanding, and specific art fluency investigation. |
| 3.22 | Specker et al. [160] | Visual art (mainly famous artworks) | Vienna Interest and Art Knowledge Questionnaire (VAIAK) | Art knowledge and expertise measure | Important addition for investigating art judgment. Used in our study to measure art expertise but not focus of our study for machine learning analysis. |
| 3.23 | Stamatopoulou [64] | Multiple art domains (poem, tapestry, play, visual art, etc.) | Aesthetic Experience Scale (AES) | 28 items, 5 factors (4–7 items each): (1) **cognitive synergies and elaboration** (e.g., to appreciate a poem more when the form enhances its meaning, to realize that the knowledge of weaving increases your enjoyment of tapestry)  (2) **emotional closeness** (e.g., to feel fulfilled when surrounded by beautiful things made by you, to like a picture because of your color preferences)  (3) **experiential emotional distancing** (e.g., to go away with a smile of pleasure when looking at an everyday scene, to forget time when participating in aesthetic activities)  (4) **paratelic mode** (e.g., to feel excited when trying to compose music or paint something, to feel emotionally enhanced and fulfilled after appreciating an artwork)  (5) **expressive perception** (e.g., to enjoy trying to identify feelings on faces in portraits, to feel completely absorbed in a work of art or music) | Interesting approach investigating aesthetic experiences. Focus on art and aesthetic preference in acting in such a context. |
| 3.24 | Virtanen et al. [161] | Images of live-world scenes (photographs) |  | Regression modeling of image quality ratings (scales and open description) leading to 68 attributes, where brightness, naturalness, and good colors were highest predictors for quality preference. | Extensive study for identifying image attribute qualities, but non-art images. |

***Note*:** In the Description section, the scales are bolded when they are adapted to the present study. Both ends of the scales are represented with different symbols, depending on the methodology used in the original studies: ‘-’ for the scales with SD (Semantic Differentiate) method (i.e., simple - complex), ‘,’ for the Likert-type scale (i.e., ugly, beautiful), ‘/’ for the binary judgement (i.e., like/dislike).

**S1 Appendix Description**

*Aesthetic aspect (I)*

The aspect of art judgments related to aesthetic evaluation encompasses two key items: (1) aesthetically moving and (2) beauty. Beauty has been a central focus in the field of art and aesthetics for centuries, with philosophers, artists, and scholars delving into its complexities between its subjective versus generalizable nature [23,24,162]. In empirical aesthetics aesthetic evaluations are the core of theoretical and empirical studies.

Beauty represents a universal and timeless judgment for valuing aesthetic appeal that has the power to captivate and evoke admiration in individuals across diverse cultures and time periods. Its allure lies in its ability to stimulate positive affective responses, pleasure, and a sense of harmony and order [163–166].

The concept of being aesthetically moved (1), also known as “being moved,” is widely recognized in the literature as a significant factor of evaluating an artwork but also as an aesthetic emotion associated with artworks [35,59,153,167]. Aesthetically moving expands our exploration beyond mere surface-level judgments of beauty and delves into the profound responses that artworks can elicit. Being aesthetically moved encompasses a range of powerful emotions experienced when engaging with art, including awe, transcendence, and wonder [99–102]. By investigating the role of being aesthetically moved in our study, we aim to capture the transformative and profound impact that art can have on individuals. In summary, the inclusion of the items aesthetically moving (1) and beauty (2) in our study is driven by their significance in capturing the essential aesthetic essence in art judgments.

*Qualitative aspects (II)*

Our investigation of qualitative aspects of art judgments encompassed three key factors: (3) good work of art, (4) creativity, and (5) technical skill. These factors aimed to capture a more universal perspective of art evaluation, focusing on constructs that are considered fundamental in determining the overall quality of an artwork [160]. Creativity (4) not only relates to the intrinsic nature of art creation as the intentional expression of something novel and unique [168], but it is also often used to assess whether an artwork is deemed valuable, particularly if it is perceived as useful for gaining, e.g., knowledge or understanding some societal or personal issue [169,170]. Therefore, creativity should be viewed in connection with the broader aspect of understanding and level of ambiguity (symbolism); while an artwork may exhibit creativity, if its meaning or associations are not comprehensible or relatable to the person’s experience, one might not grasp the intended creative thought [12].

The item “good work of art” (3) is regarded as a universal indicator of quality, with individuals providing judgments largely independent of personal preferences [3,35]. Technical skill, often considered in conjunction with creativity, refers to the level of an artist’s proficiency and expertise, and was therefore included as a qualitative aspect [171,172]. Please note, that we are providing this list considering that our study sample are non-art experts. Hence, qualitative aspects might be more diverse for art experts and is a matter of future research.

*Epistemic aspects (III)*

The exploration of epistemic aspects in art judgments particularly focused on three items: (6) fascinating, (7) interesting, and (8) thought-provoking. These items have been widely utilized in previous research to assess the level of curiosity evoked by artworks [1,36,118,119,173]. By selecting art-specific terms epistemic in nature, we aimed to capture more distinctive qualities focusing on knowledge that are strongly associated with art.

Firstly, the item fascinating (6) addresses the ability of an artwork to captivate and engage the viewer on a profound level. It implies that the artwork possesses qualities that attract and hold attention, often eliciting a sense of awe or admiration [99–101]. The concept of fascination is closely linked to the notion of being irresistibly drawn into the world depicted by the artwork, creating a sense of enchantment [102].

Secondly, the item interesting (7) reflects the degree to which an artwork arouses curiosity and stimulates intellectual engagement (Haanstra et al., 2013; Leder, 2001; Jakesch & Leder, 2009). It indicates the extent to which the artwork piques the viewer’s interest, encouraging them to explore and inquire further. An interesting artwork possesses qualities that provoke intrigue, encourage contemplation, and prompt a desire for deeper understanding.

The scale of thought-provoking (8) pertains to the artwork’s ability to provoke contemplation, reflection, and intellectual stimulation [122]. A thought-provoking artwork challenges the viewer’s preconceived notions, raises questions, and invites critical thinking [130,131]. It invites a contemplative engagement that extends beyond mere visual appreciation, encouraging viewers to reflect upon the underlying concepts, symbolism, or messages conveyed by the artwork.

*Adverse aspects (IV)*

Regarding adverse aspects of art judgments, we included two items: (9) boring and (10) disturbing/irritating. While the existing literature predominantly emphasizes hedonic experiences and positive evaluations, we aimed to address the adverse dimensions as well [59,67,175]. Understanding what makes an artwork boring (9) allows us to unravel the factors that fail to capture the viewer’s interest, resulting in disengagement and a lack of meaningful connection. By identifying the attributes associated with boredom, such as repetitive or simple motifs, predictable narratives (low symbolism), or lack of novelty, we can shed light on the aesthetic qualities that may lead to diminished engagement and reduced aesthetic appeal.

Similarly, exploring the judgments of disturbing/irritating (10) artworks offers insights into the potential boundaries and limits of aesthetic appreciation. By investigating the attributes that contribute to such adverse judgments, such as high ambiguity or lack of visual balance we gain into potential less appreciative sides of art evaluation [67, see for further reading, 68].

*Semantic aspects (V)*

The semantic aspect of art judgment encompasses the items of (11) familiarity and (12) understanding. These two items are crucial for capturing the knowledge and meaning-related dimensions of the art evaluation. According to models of aesthetic appreciation and aesthetic judgments [1,34], familiarity holds significant relevance in terms of memory and the integration of the art experience. When individuals are more familiar with an artwork, this familiarity can impact their cognitive mastery of the piece and the level of fluency in processing it [176–178]. Furthermore, familiarity as mere exposure has been found to influence judgments, such as liking [179].

Understanding the content and other elements, such as artistic style, also contributes to the creation of meaning within the artwork. This level of comprehension allows viewers to derive deeper insights and engage in the interpretation of the artwork’s underlying concepts or messages [95]. Certainly, these items have extensive relevance in an art expert group [180], yet we decided to include if for future comparison study with other cohorts. Additionally, a sense of understanding contributes to the overall art experience by facilitating a meaningful connection between the viewer and the artwork. Expanding on existing knowledge, further research has highlighted the significance of semantic processes in art judgments, shedding light on the cognitive and interpretive dimensions that contribute to the richness and depth of the aesthetic experience [e.g., 35,98].

*Preference aspects (VI)*

The personal preference aspect of art judgment, captured by the scale (13) liking, has been extensively studied in the field and remains a central focus of diverse art research inquiries (see S1 Appendix Table for list of studies using liking; note there are many more). Liking represents an essential outcome of aesthetic judgments and serves as a direct expression of personal preference [1,3]. It is considered one of the most significant indicators of an individual’s subjective response to an artwork. Despite its prominence in the literature, understanding the specific attributes that determine liking in the context of art remains a complex challenge. Researchers endeavor to unravel the intricate interplay between various factors, including visual balance, emotional resonance [59], cognitive engagement [37,94], and personal background [132,181], to gain a more comprehensive understanding of the multifaceted nature of liking as an art judgment. Hence, the exploration of art attributes and their influence on liking is crucial for many theoretical and practical implications.
