# Supplementary material for: Using machine learning to predict judgments on Western visual art along content-representational and formal-perceptual attributes
Source: PLoS One. 2024 Sep 6;19(9):e0304285. doi: 10.1371/journal.pone.0304285 (PMC11379394; doi:10.1371/journal.pone.0304285)
Supplement: S2 Appendix — (DOCX) [file pone.0304285.s008.docx]

**S2 APPENDIX**

S2.1 Appendix Table: Model comparison

|  | Gradient Boosting (GBDT) | GLM-OLS | GLM-Elastic-Net | GLM-Elastic-Net-poly-feat |
| --- | --- | --- | --- | --- |
| Avg R²  (13 judgments) | 20.27 | 19.76 | 19.83 | 20.17 |
| p-value  (paired t-test to GBDT) | - | 0.042 | 0.061 | 0.313 |

The comparison (S2.1 Appendix Table) involved the application of Generalized Linear Models (GLM) without regularization (GLM-OLS) employing the same predictors as used in the Gradient Boosting Decision Tree (GBDT) models, hence, without explicitly incorporating interaction or quadratic terms as predictors. This GLM-OLS models yielded the lowest average R² value across the 4 models under consideration. Notably, this performance was significantly inferior to that of the GBDT model.

When we employed a regularized version of the GLM, specifically the GLM-Elastic-Net, again with the same predictors as used in the GBDT, the average *R²* value was higher compared to the unregularized GLM (GLM-OLS). However, it did not reach the average R² value of the GBDT models (although the difference was not statistically significant anymore).

Further enhancing the GLM-Elastic-Net with polynomial features (specifically, 2nd degree non-linear terms encompassing interactions and quadratic terms) resulted in an additional increase in the average *R²* value. This enhanced model’s performance remained numerically but not significantly lower than that of the GBDT.

These findings underscore the value of incorporating non-linear terms in the model, as well as the benefits of regularization. Moreover, it appears that Gradient Boosting maintains a leading position in terms of predictive accuracy in our study, at least numerically.

S2.2 Appendix Table: Comparison of most important predictors between GBDT and ElasticNet linear regression

|  | Most important predictor (rank 1) | |  | Second most important predictor (rank 2) | |  | Third most important predictor (rank 3) | |
| --- | --- | --- | --- | --- | --- | --- | --- | --- |
|  | GBDT | GLM-Elastic-Net |  | GBDT | GLM-Elastic-Net |  | GBDT | GLM-Elastic-Net |
| Aesthetically moving | Emotionless vs. emotionally loaded | Emotionless vs. emotionally loaded |  | Negative vs. positive valence | Negative vs. positive valence |  | Visual harmony vs. disturbing forms | Visual harmony vs. disturbing forms |
| Beauty | Negative vs. positive valence | Negative vs. positive valence |  | Visual harmony vs. disturbing forms | Visual harmony vs. disturbing forms |  | Emotionless vs. emotionally loaded | Emotionless vs. emotionally loaded |
| Boring | Emotionless vs. emotionally loaded | Emotionless vs. emotionally loaded |  | Simple vs. complex | Simple vs. complex |  | Clear interpretability vs. symbolic | Clear interpretability vs. symbolic |
| Creativity | Clear interpretability vs. symbolic | Clear interpretability vs. symbolic |  | Emotionless vs. emotionally loaded | Emotionless vs. emotionally loaded |  | Simple vs. complex | Simple vs. complex |
| Familiarity | Negative vs. positive valence | Negative vs. positive valence |  | Loads of context vs. focused | Loads of context vs. focused |  | Visual harmony vs. disturbing forms | Visual harmony vs. disturbing forms |
| Fascinating intellectual stimulating | Emotionless vs. emotionally loaded | Emotionless vs. emotionally loaded |  | Clear interpretability vs. symbolic | Clear interpretability vs. symbolic |  | Simple vs. complex | Visual harmony vs. disturbing forms |
| Good work of art | Simple vs. complex | Simple vs. complex |  | Emotionless vs. emotionally loaded | Emotionless vs. emotionally loaded |  | Negative vs. positive valence | Negative vs. positive valence |
| Interest | Emotionless vs. emotionally loaded | Emotionless vs. emotionally loaded |  | Clear interpretability vs. symbolic | Clear interpretability vs. symbolic |  | Simple vs. complex | Simple vs. complex |
| Irritating | Negative vs. positive valence | Negative vs. positive valence |  | Clear interpretability vs. symbolic | Clear interpretability vs. symbolic |  | Realistic content vs. imaginative content | Emotionless vs. emotionally loaded |
| Liking | Negative vs. positive valence | Negative vs. positive valence |  | Emotionless vs. emotionally loaded | Emotionless vs. emotionally loaded |  | Visual harmony vs. disturbing forms | Visual harmony vs. disturbing forms |
| Technical skill | Fine brushstroke vs. rough brushstroke | Fine brushstroke vs. rough brushstroke |  | Simple vs. complex | Simple vs. complex |  | 2 dimensional vs. 3 dimensional | 2 dimensional vs. 3 dimensional |
| Thought-provoking | Emotionless vs. emotionally loaded | Emotionless vs. emotionally loaded |  | Clear interpretability vs. symbolic | Clear interpretability vs. symbolic |  | Simple vs. complex | Visual harmony vs. disturbing forms |
| Understanding | Clear interpretability vs. symbolic | Clear interpretability vs. symbolic |  | Realistic content vs. imaginative content | Realistic content vs. imaginative content |  | Negative vs. positive valence | 2 dimensional vs. 3 dimensional |
|  |  |  |  |  |  |  |  |  |

In S2.2 Appendix Table we present the three most significant predictors as identified by GBDT models and GLM-Elasti-Net linear regression for all 13 prediction targets. It is important to note that due to the intrinsic differences in the characteristics of these models (e.g., linear vs. non-linear approaches), some variations in their rankings of predictors are to be expected.

Nevertheless, our analysis reveals a remarkable consensus between these two methods regarding the two most crucial predictors. Regarding the third most important predictor, we observed minor discrepancies, limited to mere positional switches between ranks 3 and 4. For those interested in an in-depth exploration, we have uploaded comprehensive rankings of all predictors, prediction targets, and methods to our GitHub repository https://github.com/univiemops/art-rating-prediction.
