# Supplementary material for: Using machine learning to predict judgments on Western visual art along content-representational and formal-perceptual attributes
Source: PLoS One. 2024 Sep 6;19(9):e0304285. doi: 10.1371/journal.pone.0304285 (PMC11379394; doi:10.1371/journal.pone.0304285)
Supplement: Attachment — Submitted filename: Response to Reviewers_2_20240415.pdf [file pone.0304285.s010.pdf]

<u>Response to Editor & Reviewers</u>

We would like to express our gratitude to the Editor and both Reviewers for their insightful comments and reviews. We are pleased to note that Reviewer 2 is fully satisfied with the revisions we have made. Please find below our response to the comments provided by Reviewer 1.

Sincerely,

The Authors

Reviewer 1: *Thank you for your detailed response to my previous comments. Regarding the unresolved issue concerning the noise ceiling, I suggest comparing your model's performance to theoretical human-level performance. This comparison is possible given that each piece of art was evaluated by multiple participants.*

There are two potential methodologies for this comparison. The first involves predicting the average art judgments across participants, excluding the score from one held-out participant, using this held-out participant's annotation. This process should be repeated for each participant, thereby allowing the calculation of R^2 values for each iteration. Your model's performance can then be compared against the distribution of these R^2 values.

The second method assumes that the annotations follow a normal distribution, enabling you to calculate the mean and variance of art judgments for each piece using the participants' annotations. With these parameters, you could simulate human annotations by sampling from the estimated normal distribution and subsequently calculate the R^2 value against the theoretical true annotation (mean of the estimated normal distribution).

This benchmarking your model against a calculated human-level performance will provide a clearer understanding of your model's relative performance.

Reply: Thank you very much, we really appreciate your effort!

On the one hand, we would like to point out that in the paper we already present a comparison with human performance in terms of the R^2 values of the art judgement predictions (Table 3 in the manuscript).

The R^2 values quantify the errors between models and humans (which are the squared differences between single predictions of our models and the corresponding human ratings) normalized to the amount of variability of the human ratings (which is the squared difference between single human ratings and the average of human ratings) (see Figure 1).

If $\hat{y}_i$ is the predicted value of the i-th sample and y_i is the corresponding true value for total n samples, the estimated R^2 is defined as:

$$R^2(y, \hat{y}) = 1 - \frac{\sum_{i=1}^{n}(y_i - \hat{y}_i)^2}{\sum_{i=1}^{n}(y_i - \bar{y})^2}$$

where $\bar{y} = \frac{1}{n}\sum_{i=1}^{n} y_i$ and $\sum_{i=1}^{n}(y_i - \hat{y}_i)^2 = \sum_{i=1}^{n}\epsilon_i^2$.

Figure 1: Definition of the R^2 value. Source: https://scikit-learn.org/stable/modules/model_evaluation.html#r2-score

On the other hand, we agree that this measure does not allow to quantify the noise ceiling, hence, an upper bound of model performance given the consistency (or, inversely,

measurement noise) of the target variables. Unfortunately, we were not able to derive a method to make the requested comparison from your instructions above. Therefore, we turned to a library of Lukas Snoek (https://lukas-snoek.com) at github (https://github.com/lukassnoek/noiseceiling, https://doi.org/10.5281/zenodo.7233867) to make the computations. This library computes the maximum possible R^2 score given the variance in the dependent variable. For the results see Table 1.

For most of the art judgments our GBDT models performed better than the theoretical noise ceiling limits. However, this surprising result can be explained by the lack of true repeated measures in our data. The repeated measures used here come from different subjects. Thus, although each subject rated the same picture with respect to a particular art-judgement, each subject also rated the art-attributes of the picture accordingly. Hence, our GBDT models were able to capture relationships between art-attributes and art-judgments (as intended) and to surpass the computed theoretical limits, because parts of the art-judgement variability can be explained by the art-attributes. Hence this noise ceiling approach is not well suited in our specific case. It would be well suited if the same subjects would have made repeated judgements.

Therefore, we don't see how this comparison is informative for readers and we wouldn't like to include it in the paper. However, we remain open to any suggestions on this matter.

Table 1: Comparison of observed model performance and theoretical noise ceiling.

**Scales of art-judgments**	**GBDT observed model performance: R^2 ± SD**	**Noise ceiling: upper bound of model performance: R^2 ± SD**
Aesthetically moving	0.15 ± 0.04	0.08 ± 0.01
Beauty	0.20 ± 0.06	0.16 ± 0.03
Good work of art	0.14 ± 0.06	0.08 ± 0.01
Creative	0.31 ± 0.04	0.29 ± 0.03
Technical skill	0.16 ± 0.09	0.12 ± 0.01
Fascinating (intellectual)	0.22 ± 0.04	0.18 ± 0.02
Interesting	0.25 ± 0.04	0.19 ± 0.02
Thought provoking	0.22 ± 0.05	0.18 ± 0.02
Boring	0.22 ± 0.05	0.17 ± 0.02
Disturbing, irritating	0.32 ± 0.05	0.37 ± 0.03
Familiarity	0.02 ± 0.04	0.22 ± 0.07
Understanding	0.24 ± 0.08	0.21 ± 0.03
Liking	0.18 ± 0.05	0.12 ± 0.02
