# Supplementary material for: Using machine learning to predict judgments on Western visual art along content-representational and formal-perceptual attributes
Source: PLoS One. 2024 Sep 6;19(9):e0304285. doi: 10.1371/journal.pone.0304285 (PMC11379394; doi:10.1371/journal.pone.0304285)
Supplement: Attachment — Submitted filename: Response to Reviewers_20240229.docx [file pone.0304285.s009.docx]

**Response to Editor & Reviewers**

We are encouraged by the positive reception of our paper in its initial iteration and are optimistic that the revisions, guided by your feedback, have further refined and improved the manuscript.

Below, you will find our responses to each of the comments and suggestions raised. To ensure clarity and coherence, we have organized the reviewers' feedback into paragraphs, categorizing them conceptually. This is followed by our detailed reply, including specific references to the corresponding sections in the revised manuscript where in-text changes have been made.

In the revised manuscript, we have highlighted all major changes in red text for ease of identification. Additionally, we have undertaken a thorough review of the entire document, correcting language errors and typographical mistakes to enhance its readability and accuracy.

We thank you once again for your invaluable contributions. We eagerly look forward to continuing our collaboration towards the successful publication of this work.

The authors

**General changes:**

In response to your comments, particularly from Reviewer 2, we have dedicated additional time to meticulously proofread the entire manuscript. Our focus during this thorough review was primarily on optimizing the language and enhancing the overall clarity of the text.

**Reviewer 1:**

**Reviewer 1:** *The authors of this paper have conducted a comprehensive review to identify the key attributes of arts and art judgments categories. They employed a nonlinear machine learning technique to demonstrate that behaviorally collected art judgments can be predicted using these key attributes, which were also gathered behaviorally. Additionally, the authors analyzed the contributions of each attribute to the prediction of various categories of art judgments.
The study exhibits a meticulous approach to the literature review and effectively applies machine learning methodology. However, some crucial questions remain to be addressed.*

**Reply:** We sincerely appreciate your thoughtful comments. Rest assured, we are fully committed to addressing the remaining questions and concerns raised.

**Reviewer 1:** *It is imperative to conduct a model comparison with other models to substantiate the authors’ findings and establish the necessity of nonlinearity in their approach. Specifically, I propose a comparative analysis that includes a GLM model incorporating attributes, their interactions and their quadratic terms to predict judgments, in order to gauge the superiority of the GBDT model.*

**Reply**: We appreciate your suggestion, please find the requested comparison below.

Table 1: Model comparison

|  | Gradient Boosting (GBDT) | GLM-OLS | GLM-Elastic-Net | GLM-Elastic-Net-poly-feat |
| --- | --- | --- | --- | --- |
| Avg R² (13 judgments) | 20.27 | 19.76 | 19.83 | 20.17 |
| p-value (paired t-test to GBDT) | - | 0.042 | 0.061 | 0.313 |

The comparison (Table 1, added in the manuscript in Appendix B) involved the application of Generalized Linear Models (GLM) without regularization (GLM-OLS) employing the same predictors as used in the Gradient Boosting Decision Tree (GBDT) models, hence, without explicitly incorporating interaction or quadratic terms as predictors. This GLM-OLS models yielded the lowest average R² value across the 4 models under consideration. Notably, this performance was significantly inferior to that of the GBDT model.

When we employed a regularized version of the GLM, specifically the GLM-Elastic-Net, again with the same predictors as used in the GBDT, the average R² value was higher compared to the unregularized GLM (GLM-OLS). However, it did not reach the average R² value of the GBDT models (although the difference was not statistically significant anymore).

Further enhancing the GLM-Elastic-Net with polynomial features (specifically, 2nd degree non-linear terms encompassing interactions and quadratic terms, as requested) resulted in an additional increase in the average R² value. This enhanced model's performance remained numerically but not significantly lower than that of the GBDT.

These findings underscore the value of incorporating non-linear terms in the model, as well as the benefits of regularization. Moreover, it appears that Gradient Boosting maintains a leading position in terms of predictive accuracy in our study, at least numerically.

While we acknowledge the appeal of such additions, we would like to elucidate our rationale for not incorporating them in the main body of the submission.

In our assessment, there exists a sufficient corpus of high-quality model comparisons within the extant literature. Introducing yet another comparative analysis, in our estimation, may not contribute substantially to the existing body of knowledge. Our primary objective with this manuscript is to illustrate the practical application of innovative methodologies to noteworthy problems, thereby offering insights into their real-world utility.

Given the comprehensiveness of the present paper, we are concerned that the inclusion of an additional model comparison could potentially result in an intricate and overloaded presentation. Our intention is to maintain clarity and conciseness. Hence, we opt for including this comparison in the appendix of the manuscript.

We appreciate your understanding of our perspective and remain open to further discussion on this matter.

**Reviewer 1:** *Additionally, it would be valuable to assess the significance of each attribute in predicting judgements by comparing with other feature selection methodologies, such as ridge regression. Such comparisons are necessary to validate the claims made in this study regarding the importance of individual attributes. For example, the disparities in finding between this paper and Iigaya’s work, where the significance of concreteness (equivalent to abstraction in this work) and valence yield opposing results (figure 1 and 3). These discrepancies, albeit stemming from varying features, stimuli, and methodologies, necessitate comparisons with other models for robust validation.*

**Reply:**	Thank you very much for your valuable feedback! We concur with your perspective on the utility of converging evidence from multiple models. In Table 2 (added in the manuscript in Appendix B) we present the three most significant predictors as identified by GBDT models and GLM-Elasti-Net linear regression for all 13 prediction targets. It is important to note that due to the intrinsic differences in the characteristics of these models (e.g., linear vs. non-linear approaches), some variations in their rankings of predictors are to be expected.

Nevertheless, our analysis reveals a remarkable consensus between these two methods regarding the two most crucial predictors. Regarding the third most important predictor, we observed minor discrepancies, limited to mere positional switches between ranks 3 and 4. For those interested in an in-depth exploration, we have uploaded comprehensive rankings of all predictors, prediction targets, and methods to our GitHub repository <https://github.com/univiemops/art-rating-prediction>. In balancing the acquisition of knowledge with the clarity of presentation, we are convinced that incorporating these detailed rankings into the main body of the manuscript might not substantially enhance its value and could potentially detract from its conciseness. Hence, we opt again for putting them into the appendix (incl. the github link to all details). We appreciate your understanding of our rationale in this regard and are open to further discussion should there be additional aspects of this matter you wish to explore.

Table 2: Comparison of most important predictors between GBDT and ElasticNet linear regression

|  | Most important predictor (rank 1) | |  | Second most important predictor (rank 2) | |  | Third most important predictor (rank 3) | |
| --- | --- | --- | --- | --- | --- | --- | --- | --- |
|  | GBDT | GLM-Elastic-Net |  | GBDT | GLM-Elastic-Net |  | GBDT | GLM-Elastic-Net |
| Aesthetically moving | Emotionless vs. emotionally loaded | Emotionless vs. emotionally loaded |  | Negative vs. positive valence | Negative vs. positive valence |  | Visual harmony vs. disturbing forms | Visual harmony vs. disturbing forms |
| Beauty | Negative vs. positive valence | Negative vs. positive valence |  | Visual harmony vs. disturbing forms | Visual harmony vs. disturbing forms |  | Emotionless vs. emotionally loaded | Emotionless vs. emotionally loaded |
| Boring | Emotionless vs. emotionally loaded | Emotionless vs. emotionally loaded |  | Simple vs. complex | Simple vs. complex |  | Clear interpretability vs. symbolic | Clear interpretability vs. symbolic |
| Creative | Clear interpretability vs. symbolic | Clear interpretability vs. symbolic |  | Emotionless vs. emotionally loaded | Emotionless vs. emotionally loaded |  | Simple vs. complex | Simple vs. complex |
| Familiarity | Negative vs. positive valence | Negative vs. positive valence |  | Loads of context vs. focused | Loads of context vs. focused |  | Visual harmony vs. disturbing forms | Visual harmony vs. disturbing forms |
| Fascinating intellectual stimulating | Emotionless vs. emotionally loaded | Emotionless vs. emotionally loaded |  | Clear interpretability vs. symbolic | Clear interpretability vs. symbolic |  | Simple vs. complex | Visual harmony vs. disturbing forms |
| Good work of art | Simple vs. complex | Simple vs. complex |  | Emotionless vs. emotionally loaded | Emotionless vs. emotionally loaded |  | Negative vs. positive valence | Negative vs. positive valence |
| Interest | Emotionless vs. emotionally loaded | Emotionless vs. emotionally loaded |  | Clear interpretability vs. symbolic | Clear interpretability vs. symbolic |  | Simple vs. complex | Simple vs. complex |
| Irritating | Negative vs. positive valence | Negative vs. positive valence |  | Clear interpretability vs. symbolic | Clear interpretability vs. symbolic |  | Realistic content vs. imaginative content | Emotionless vs. emotionally loaded |
| Liking | Negative vs. positive valence | Negative vs. positive valence |  | Emotionless vs. emotionally loaded | Emotionless vs. emotionally loaded |  | Visual harmony vs. disturbing forms | Visual harmony vs. disturbing forms |
| Technicalability | Fine brushstroke vs. rough brushstroke | Fine brushstroke vs. rough brushstroke |  | Simple vs. complex | Simple vs. complex |  | 2 dimensional vs. 3 dimensional | 2 dimensional vs. 3 dimensional |
| Thoughtfulness | Emotionless vs. emotionally loaded | Emotionless vs. emotionally loaded |  | Clear interpretability vs. symbolic | Clear interpretability vs. symbolic |  | Simple vs. complex | Visual harmony vs. disturbing forms |
| Understanding | Clear interpretability vs. symbolic | Clear interpretability vs. symbolic |  | Realistic content vs. imaginative content | Realistic content vs. imaginative content |  | Negative vs. positive valence | 2 dimensional vs. 3 dimensional |
|  |  |  |  |  |  |  |  |  |

**Reviewer 1:** *While the paper demonstrated the importance of individual attributes, it falls short of elucidating how the manipulation of these attributes impact art judgments in an interpretable manner. I recommend conducting a comprehensive analysis of the GBDT model’s predictions while varying input attributes, as this would provide insight into the relationship between the art attributes and the art judgments. (e.g., how the liking rating changes when the abstraction level goes up)*

**Reply:** We appreciate your thoughtful feedback! We did these computations based on SHAP values already before submission but did not include them in the manuscript for the sake of clarity and space constraints. The detailed results are available in our GitHub repository at <https://github.com/univiemops/art-rating-prediction>. The specific example you referenced, illustrating the fluctuation in liking ratings with changes in abstraction levels, is shown below.

Figure 1: How is liking rating influenced by abstraction.

Regrettably, due to constraints imposed by space limitations and the imperative to maintain the clarity of our paper, we refrained from presenting the exhaustive details of our findings, encompassing 17 predictors across 13 predictions, resulting in a total of 221 plots in the manuscript. However, we added the link to the github repository to make our detailed results available. Note, by providing all plots in the repository, we cut Tables A2-A4 in Appendix A). Nevertheless, we remain receptive to further dialogue on this.

**Reviewer 1:** *Furthermore, it would be beneficial to include calculations for the noise ceiling to assess the theoretical upper limit for predicting art judgement based on the attributes. This evaluation will offer a deeper understanding of the model’s capabilities and the limit of the machine learning based approach in this literature.*

**Reply:** Thank you very much for your feedback! Building upon Lage-Castellanos et al.'s (2019) approach, we explored adapting their noise ceiling computation for fMRI to our machine learning framework. However, the inherent differences between neuroimaging and our data render direct application impractical. While we investigated several alternative methods for estimating theoretical prediction limits, like intra class correlation (Shrout 1979), none offered a feasible solution. We are open to exploring other options and would be grateful if you could share any specific methods you have in mind that might be more suitable for our data and framework.

Lage-Castellanos A, Valente G, Formisano E, De Martino F (2019) Methods for computing the maximum performance of computational models of fMRI responses. PLoS Comput Biol 15(3): e1006397. <https://doi.org/10.1371/journal.pcbi.1006397>

Shrout, P. E., & Fleiss, J. L. (1979). Intraclass correlations: uses in assessing rater reliability. Psychological bulletin, 86(2), 420.

**Reviewer 1:** *In conclusion, although this research is meticulously designed and executed, addressing the aforementioned points will enhance the study’s robustness.*

**Reply:** We appreciate the acknowledgment of the meticulous design and execution of our research. Thank you for guiding us toward further refinement and improvement.

**Reviewer 2:**

**Reviewer 2:** *I have reviewed this paper and find it highly interesting and well done. I have however a number of recommendations that would make the paper better. Even though none of my concerns were large enough to warrant the answer "No" to questions 1-4, they are together larger than warranting just cosmetic changes, and thus my recommendation becomes "Major revision".*

**Reply:** We sincerely appreciate your positive evaluation of our work, and we are grateful for the time and effort you have invested in reviewing it. We agree on your recommended changes, and we are committed to addressing your concerns to enhance the quality of the paper.

**Reviewer 2:** *The topic of the paper, to investigate the correlations between subjective attributes of an artwork and value-based judgements of the same artwork, is highly interesting and relevant. A data-driven method based on recordings of user studies, and a regression-based Machine Learning (ML) method has been used. The method is clearly described and reproducable, and the results are rigorously analyzed and discussed. All conclusions in the (extremely ambitious and rigorous) Discussion section are well underbuilt by the experimental results.
Recommendation:
With a background in ML, I have some suggestions. I firstly would like to emphasize that I do not ask for new or changed experiments, as the currently applied ML method is adequate for your study.
However, I have requests concerning the framing and references to the state of the art. I recommend a newer ML introduction reference than Breiman, e.g. Alpaydin's book from 2020: <https://ds.amu.edu.et/xmlui/bitstream/handle/123456789/6943/Introduction%20to%20Machine%20Learning.pdf>. In general, this field is moving forward so fast that I recommend you to check with Alpaydin's book if other ML references in your list are outdated, e.g. Cawley and Talbot.*

**Reply:** Thank you very much, we added

Alpaydin, E. (2022). Maschinelles Lernen. Berlin, Boston: De Gruyter Oldenbourg. https://doi.org/10.1515/9783110740196

to our references and cited it where appropriate (e.g., page 7).

**Reviewer 2:** *You should also motivate why you are using a Random Forest method when there are more modern Deep Learning methods that are more powerful. (The answer is that with your limited dataset, a Random Forest method is more appropriate so you have chosen the right method - but if you had more data it would indeed be a better idea to use a neural network which can capture more nonlinear relationships.)*

**Reply:** Thank you for your insightful comment! Indeed, we opted for gradient boosted decision tree models instead of Random Forests, as explicitly stated in our methods section. Our model selection was primarily driven by the findings of recent research, such as the work by Grinsztajn et al. (2022), which suggests that tree-based models often outperform deep learning on typical tabular data like ours. We added this note in our Methods section (page 17). We hope this additional information clarifies our model choice and demonstrates a robust justification based on current research.

Grinsztajn, L., Oyallon, E., & Varoquaux, G. (2022). Why do tree-based models still outperform deep learning on typical tabular data? In Advances in Neural Information Processing Systems (Vol. 35, pp. 507-520).

**Reviewer 2:** *The statistical analysis is very carefully designed and builds on a selection of relevant recent work.*

*All the data (i.e. the individual answers by each test person) are made available for experimental comparison.*

*The language is indeed good overall, and the manuscript is easy to follow.*

*However, I would recommend to let a native English speaker go over the manuscript text. There are numerous Germanic language constructions with very long sentences, that could be divided up. This would make the text easier to read. There are also some convoluted sentences that are difficult to understand, e.g.
p30: "If we were successful ... artwork evaluation."
p31: "The ability of artworks ... Wagemans, 211b)."
p34: "By employing machine ... interesting ones."*

**Reply:** We appreciate your feedback on the language and readability of our manuscript. We agree that there may be instances of lengthy sentences and convoluted phrases that were restructured for improved readability. We engage a native English speaker to thoroughly review and refine the manuscript. We hope to have enhanced the overall clarity of the manuscript to ensure that our research is easily accessible also to a wider audience.
